# The Impact of Swimming on Fundamental Movement Skill Development in Children (3–11 Years): A Systematic Literature Review

**DOI:** 10.3390/children10081411

**Published:** 2023-08-19

**Authors:** Lauren Sinclair, Clare M. P. Roscoe

**Affiliations:** Department of Sport and Exercise Science, Clinical Exercise and Rehabilitation Research Centre, University of Derby, Kedleston Road, Derby DE22 1GB, UK; l.sinclair1@unimail.derby.ac.uk

**Keywords:** swimming, fundamental movement skills, motor development, children, aquatic, locomotor, object control, learn to swim, curriculum

## Abstract

Swimming is the only sport providing lifesaving skills, reducing the risk of death by drowning, a top cause of deaths in children aged 1–14 years. Research shows swimming amongst other sports can aid fundamental movement skill (FMS) development. Therefore, this review investigated the following: (1) how swimming impacts FMS development in children aged 3–11 years, (2) successful tools assessing swimming and FMS, and (3) recommendations appropriate to the UK curriculum based on findings of this study. A systematic literature review using Google Scholar, PubMed, and SPORTDiscuss was conducted to investigate the effects of swimming on FMS development. Methods included database searching, finalising articles appropriate to the inclusion and exclusion criteria, and identifying relevant articles using the Preferred Reporting Items for Systematic Reviews and Meta-Analyses. The Mixed Methods Appraisal Tool assessed data quality and bias risk, whilst thematic analysis synthesised data alongside descriptive results. Ten papers were synthesised, identifying significant positive impacts of swimming on FMS, including significant pre–post testing (*p* < 0.01), significant improvements compared to other sports (*p* < 0.001), and significant improvements in specific motor skills (Balance; *p* = 0.0004). Future research specifically addressing swimming and FMS is essential to improving the curriculum.

## 1. Introduction

Current research on fundamental movement skills (FMS) and the impact of swimming is limited, with research considering the effects of swimming often being incorporated alongside other sports like football [1], gymnastics [2], and general physical activity (PA) [3] and in children with disabilities [4], which does not focus on swimming-specific effects on FMS development. Those limited papers that do address the effects of swimming on FMS can be dated, based overseas [5,6], or based on different curriculum and assessment guidelines or unreliable swimming assessment methods [7]. Previous research indicates that swimming intervention can improve FMS development [1], reduce stress and overstimulation in children with disabilities [4], and make joint manipulation easier due to reduced weight bearing.

Although limited, research supports the positive impact of swimming intervention on FMS development [8,9] and highlights a need for a more modern and universal swimming assessment tool [10]. This is particularly true for UK research, representative of the population, in addressing the importance of swimming within the UK curriculum and Swim England (SE; [11,12,13]). Research supporting the general benefit of swimming to FMS is non-specific, with research generalising swimming rather than breaking it down into swimming-specific skills, highlighting a gap in the literature looking at specific effects of swimming on FMS in children [14].

### 1.1. Swimming

Swimming can improve general health, mental health [15], cardiovascular endurance, muscular strength, flexibility, coordination, balance, and much more [16], but it also allows for children to interact with new people of a similar ability to themselves, develop new skills, and socialise [17]. Swimming is a highly inclusive sport and can be for all ages, sizes, ethnicities, and backgrounds; children or adults with disabilities can also participate in the sport, which is extremely beneficial to their wellbeing [18]. Swimming is the only sport that is a lifesaving skill [17] and is often seen as an essential life skill and sport for this reason. Currently, swimming is the only compulsory sport in the UK national curriculum [17], with schools having to provide lessons in key stage 1 or 2 (KS1 or KS2; [13]), which encourages pupils’ time in the water; however, the purpose of this is to provide general water safety such as swimming competently for 25 m and performing self-rescue [18], not to aid FMS development specifically. The government provides schools with a PE Sports Premium, used to provide equipment, lessons, and training for staff in order to deliver high-quality PE lessons in primary schools across the UK [19], with guidance suggesting where schools should allocate money to, which may be why only 53% of KS1/2 students access swimming lessons [11]. According to recent government statistics [14], local authority schools (LAS) were allocated GBP 6970.00 per pupil for the 2022–2023 academic year (GBP 6780.00 for 2021–2022), for pupils aged 5–16 years. This money is for employing staff, maintenance, equipment, and supplies. In the 2021–2022 academic year, total expenditure for UK LAS was GBP 23.1 billion [20], only 13% was spent on supplies including sports equipment. For the 2021–2022 academic year, school sports premium (SSP) funding provided five goals for primary schools to work towards [14,19], including increasing attainment and achieving swim competency by the end of KS2; however, this was scarcely achieved: one in four students did not reach swim competency, with only 34% meeting full competency criteria [11]. Again, the SSP provides guidance rather than policy, which allows schools to spend funds as they wish [21] and could potentially disadvantage some children who then do not access swimming lessons regularly. Guidance is provided by the government [14] and Active Partnerships [22], an organisation supported by the government to advise schools on spending which could be a contributing factor to why not all KS2 children achieve swim competency post-KS2 [14].

In recent years, the emergence of new assessment tools for swimming includes the aquatic movement protocol (AMP) [10], backed by the increasing number of children and adults dying from drowning, a preventable cause of death. In 2021, the World Health Organisation (WHO) [23] identified that drowning is a top-five cause of deaths in 1–14-year-olds in 48 of 85 countries worldwide, highlighting the significance of learning swimming not just for FMS development, but also as a lifesaving tool. The AMP [10] is one of the first tools proposed to effectively assess aquatic motor competence in young children. Whilst this study identified a significant relationship (*p* = 0.01) between swimming and improved FMS, there needs to be further research to assess the test–retest reliability of the AMP, which may prove to be a useful tool that can be utilised by swimming coaches, education teachers, and sport scientists [10], especially in the UK where research is limited.

### 1.2. Fundamental Movement Skills

Motor development is the change or improvement in motor skills (MS); FMS competency is a prerequisite to daily functioning and participation in PA or sport-specific activities [24], primarily developed in pre-school-aged children (3–5 years) [25], a critical time for FMS development influenced by instruction and practice [26]. If a child were to not develop specific locomotor and object control skills during pre-school years, they can become limited in MS ability. Research highlights that FMS delays can mirror inactivity in adolescence [25,27], making everyday tasks harder, and contributes to poor coordination and motor function which are essential to daily routines in adulthood.

Exercise is known to improve brain function, cognition [28,29], and coordination [30] as well as develop locomotor control, the ability to move through different environments using movements including running, walking, hopping, skipping, and jumping, for example [31], whereas object control involves skill and control of something external from the body such as kicking a ball, catching a netball, hitting, throwing, and striking [32]; a child’s ability to do these things increases their activity participation and academic achievement likelihood in later life [33], whereas children who are underdeveloped in these areas are associated with higher risk of inactivity and sedentary behaviour [34], with inactivity due to poor FMS being more common in children with disabilities too.

Research surrounding developing FMS is often linked to assessment methods like the Test of Gross Motor Development-2 (TGMD-2) which is highly reliable for testing children’s gross motor development between 3–11 years of age [35]; the TGMD-2 uses two subtests for locomotor and object control, making it a highly appropriate assessment method for FMS in primary school-aged children, using locomotive and object manipulative skills relative to this age group. This test is easily used by a range of professionals, which means teachers could use this to look at the progression of their students, as well as in research settings [36], as research has identified the reliability and consistency of the TGMD-2, distinguishing between both fine and gross MS [37]. However, the TGMD-2 does have its limitations, as it does not take into account other factors such as special educational needs and disability (SEND) and personal and environmental factors [38]. Additionally, the Bruininks–Oseretsky Test of Motor Proficiency (BOTMP) [39] uses products to assess gross and fine MS, similar to the TGMD-2 but with more specific tests, which limits the age range the tests are suitable for; further, it only provides generalised texts for motor proficiency and does not distinguish between gross and fine MS [36]. Both BOTMP and TGMD-2 provide reliable and valid results; however, they only assess FMS on land, without consideration for transferable skills learnt in the water, such as kicking, jumping, throwing, and balance, which are shown to develop through swimming [16]. In addition, the BOTMP can be used to assess children with disabilities [39], whereas the TGMD-2 does not take factors such as SEND and environment into account [38], which is significant when the number of children in the UK living with a SEND is increasing: from 6% of children in 2010 [40] to 9% in 2022 [41].

All presented publications from the literature highlight the requirement for children in KS1 and KS2 to be able to swim competently and to be aware of safety issues around water to prevent children from drowning (ages 1–14 years). FMS is primarily developed in pre-school children (3–5 years) as previously stated, with FMS testing being highly utilised in children aged 3–11 years. The authors of this study felt that with all the characteristics associated with improving swimming and FMS competency, it was appropriate to concentrate on pre-school children (3 years) and children reaching the end of primary school/KS2 in England (11 years). Therefore, with this justification of age, the following aims of this literature review are (1) to assess if there is an impact of swimming on FMS development in children aged 3–11 years; (2) to identify successful tools that assess swimming and FMS individually; and (3) to conclude with recommendations appropriate to the UK curriculum based on the findings of this study.

## 2. Materials and Methods

### 2.1. Protocol and Registration

The systematic literature review (SLR) was registered on PROSPERO on 22 March 2023 (registration number CRD42023384361). The SLR protocol is available on the PROSPERO website when searching the registration number, which is alternatively found by using the link: https://www.crd.york.ac.uk/prospero/display_record.php?ID=CRD42003384361, accessed on 22 March 2023.

### 2.2. Criteria for Study Selection

The literature was systematically reviewed, using the Preferred Reporting Items for Systematic Reviews and Meta-Analyses (PRISMA) [42] and updated framework [43], to narrow down works in the literature published between January 2008 to December 2022 (15-year span), identifying all peer-reviewed, English language articles analysing the effect of swimming on FMS in children aged between 3–11 years, reflective of predominately primary school-aged groups [13]. English language articles may originate from across the world, therefore showing a broader reflection of the effect of swimming on FMS and the importance of this for children. Within the search, all experimental, primary data-based studies were included (random and non-random control trials, experimental, observational, cohort studies); review articles were excluded.

Articles examining the effect of swimming on FMS met the following series of criteria: participants 3–11 years; swimming measured either observationally or using a form of aquatic movement assessment such as the AMP [10]; having used an instrument to evaluate FMS; primary data collection through baseline or observational study; peer-reviewed; analysing swimming and FMS or variant terminology. Articles wherein participants were outside of the 3–11-year age bracket were excluded to keep analysis predominately within the primary school age bracket, as swim competency should be achieved by KS2 [13]. Articles exceeding the 3–11 age bracket, but whose mean age was within the bracket, were included. Articles wherein data needed for data extraction (e.g., participant age) were missing were excluded to ensure accurate data collection. Studies including participants with special educational needs and disability (SEND) were included if they met the inclusion and exclusion criteria, due to limited research, thereby promoting inclusivity by assessing a variety of participants in terms of ability. Studies using only qualitative data were excluded; studies which use both qualitative and quantitative data were included, due to the lack of research available. Finally, studies which address sports in addition to swimming (e.g., football) [1] were included, providing outcome data availability and relevance.

### 2.3. Search Strategy

**Figure 1:**
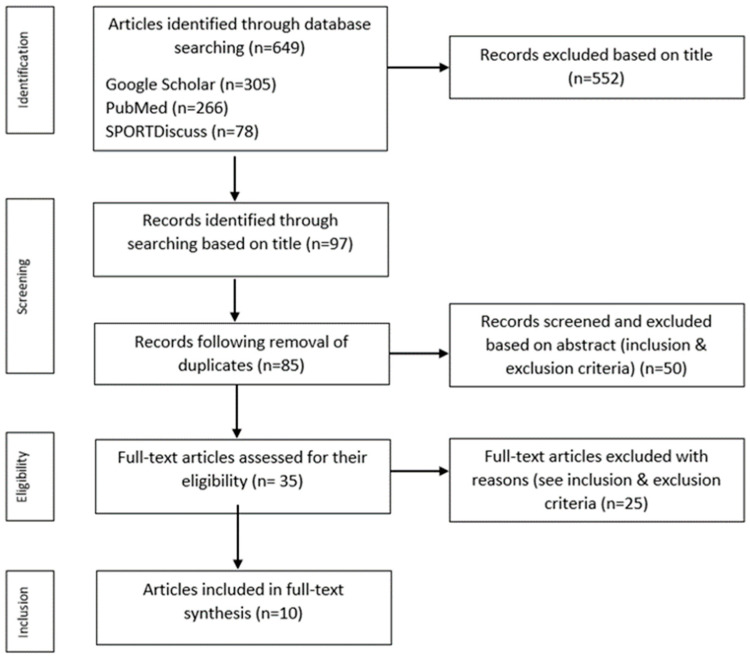
Combined searches following the PRISMA flow chart regarding papers found using the search: “Allintitle: *fundamental movement skills* *swimming* *children*” [42].

Google Scholar, SPORTDiscuss, and PubMed/Medicine were searched up to December 2022 using the following key words: fundamental movement skills, swimming, and children. For example, PubMed was searched using the following: “Allintitle: *fundamental movement skills* *swimming* *children*”. As both swimming and FMS can have alternative accepted terminology, for swimming, terms included the following: swimming; swimmer; swim; swim skills; swimming proficiency; aquatic movement; aquatic skills; aquatic motor skills; aquatic competence; aquatic readiness; aquatic environment; water skills; water safety; water therapy; water movement; drowning; and drowning prevention. For FMS, terminology included the following: fundamental movement skills; fundamental motor skills; foundational movement skills; gross motor skills; gross motor function; motor skills; motor ability; motor coordination; motor competence; motor competency; motor learning; motor performance; motor development; motor proficiency; locomotor skills; and object control. Titles were screened according to the criteria and duplicated papers from different search engines were subsequently removed. An additional screening of the abstract was undertaken and, in the case of uncertainty as to whether inclusion criteria had been fulfilled, the article was included in the full-text screen. Full-text articles were reviewed for eligibility. The search strategy was completed by the lead researcher (LS) and may be viewed in Figure 1. The original search sample was later shared with the second researcher (CR) to ensure agreement on the inclusion of studies. For studies that were not initially agreed upon, a discussion was held to reach a mutual decision on the inclusion of specific articles. A final search was carried out prior to write up to check for new updates since the initial search.

### 2.4. Data Extraction Synthesis

Articles that met all criteria were then input into a table to log relevant information including author(s); date of publication; country of study; the setting of the study (field (a school), laboratory); sample size; sample age range; participants’ mean age; the study design; the outcome measure; and the overall findings of swimming and FMS. Outcome measures looked at FMS assessment through tests such as the TGMD-2; swimming competency was reported observationally or using testing methods like the AMP [10]. 

### 2.5. Study Quality Assessment

Due to the variability of study design, the Mixed Methods Appraisal Tool (MMAT) [44] was the most appropriate method of assessing the quality and bias risk of the studies finalised for review. The MMAT used 2 initial questions used for all papers regardless of study design, followed by 5 assessment questions relevant to the study design; for this study, these questions were from either quantitative randomised controlled trials or quantitative non-randomised trials. Answers to each question were scored, up to a maximum of 7 points, for 7 questions. Questions answered with yes received 1 mark and questions answered with cannot tell or no were awarded no marks; therefore, a high score indicated the high quality of the study, whereas a low mark would indicate risk of bias or poor quality. Scores following the MMAT can be found in Table 1. Whilst the MMAT indicated the quality assessment of each paper, it did not contribute to the inclusion and exclusion criteria.

### 2.6. Analysis

Due to the lack of research specifically regarding the effects of swimming on FMS, the analysis of collated articles is of a narrative approach. Thematic analysis assessed factors within the research question from a qualitative perspective, paired with the quantitative pre–post data of each study. The thematic analysis followed Braun and Clarke’s method which aims to identify patterns across a data set relating to the benefits of swimming on FMS development [49,50]. Thematic analysis is comprised of the following 6 phases [50]: (1) familiarisation with data, reading all transcripts and data presented, (2) generating codes from the data/text presented, (3) searching for themes in the codes (usually, there are multiple themes and sometimes subthemes which fall under those themes), (4) reviewing themes, making sure all codes have been assigned to a theme, (5) defining and naming themes that are easily understood, and (6) reporting. This analysis can either be guided by existing theories as well as the researcher’s standpoint and subject knowledge (theoretical) or led by coding without trying to fit the data into a mould or pre-existing frame (inductive). Both are accepted forms of analysis; inductive thematic analysis does reduce the influence of the researcher’s standpoint, although not completely [49]. For this study, a combination approach was used [51], due to there being a lack of previous research on this topic; therefore, there was no mould to fit into, reducing the risk of bias from the researcher’s standpoint [51] and allowing the data to shape themes truthfully and be easily interpretable [52].

## 3. Results

### 3.1. Study Selection

Using PRISMA methods (Figure 1), a total of 649 papers were found in February 2023 using the search “Allintitle: *fundamental movement skills* *swimming* *children*” on three search engines (Google Scholar, SPORTDiscuss, PubMed/Medicine). A total of 552 papers were excluded based on their title for reasons including, but not limited to, irrelevancy or not being available in English language, for example. The remaining 97 papers were reduced to 85 after removing duplicates; abstracts were then analysed and excluded based on available criteria, resulting in 50 papers failing to meet the inclusion criteria. In instances where it was unclear if the paper met the inclusion/exclusion criteria, they were included for full-text analysis. Full-text analysis included 35 papers, where 25 were removed for reasons including (but not limited to), mean age failing to meet inclusion criteria, no data specific to swimming and FMS, and no pre–post data. As a result, only 10 papers met the inclusion/exclusion criteria and were, therefore, included in full-text analysis (Table 2). Figure 1 demonstrates the process carried out [42].

### 3.2. Origin and Participants

Of the final 10 studies, only 2 originated in the UK [7,10]. The remaining eight originated from various countries, including Portugal [8,9,48], Serbia [6,45], Romania [47], Poland [46], and Turkey [5]. All studies reported gender and age as required by the inclusion/exclusion criteria; participants of the studies were predominantly male: out of a combined total of 611 participants from all studies, 429 (70.2%) were male, and only 182 (29.8%) were female. Ideally, it would be more representative to have equal male and female participants; however, this is often not possible. Participants had an average age of 7.7 years, fitting with UK primary curriculum recommendations [13] for swimming lessons, as well as being in the middle of this review’s target population age bracket.

### 3.3. Study Design

All ten of the articles included were forms of randomised controlled trials. Three of the articles were classed as randomised controlled trials and an additional seven were classed as non-randomised. Of these, one had an observational longitudinal element to it.

### 3.4. Study Quality Assessment

The MMAT [44] assessed the quality of studies due to the variance in methods used in each study. All studies scored high, with independent scores reported in Table 1. Collectively, all studies met five or more of the criteria out of a possible seven set out by the MMAT criteria [44]: 30% of studies scored highly (seven of seven), 50% met six of the criteria, and 20% met five of the criteria. When assessing each study using the MMAT tool, three studies fell into category 2 (quantitative randomised controlled trials) and seven fell into category 3 (quantitative non-randomised). Due to all studies containing all required data needed to meet the inclusion criteria, there were no missing data and, therefore, there was no need to assess risk of bias due to missing results.

### 3.5. Swimming and FMS Development Articles

Ideally, all studies analysed would have utilised the same testing methods of swimming and FMS; however, limited research surrounding this topic and limited robust assessment tools for swimming meant this was not possible [10]. As a result, a meta-analysis was not possible and, therefore, analysis was of a thematic nature [50], to ensure a meaningful analysis was carried out. Swimming assessments included observations, AMP [10], or Water Orientation Test Alyn 2 (WOTA-2) [53]. In addition, there is no standalone FMS assessment tool; researchers used a variety of methods including the TGMD-2 [35], Gross Motor Function Measurement—88 Tests (GMFM-88) [54], Körperkoordinationtest Für Kinder test (KTK) [55], EUROFIT [56] tests, and Standardized Movement Assessment Battery (SMAB) [57]. With this in mind, the inclusion/exclusion criteria could not specify the type of assessment battery used as there is so much variability in testing methods as well as lack of research in this field generally. All test methods were used twice except for the AMP and SMAB (used once) and TGMD-2 (three times). The AMP was used once as the study was testing its robustness in assessing aquatic motor skill development [10], whereas the TGMD-2 is a more common and reliable test for FMS [35]. It is likely the SMAB was used less often due to being dated [57], and the study using this test method was aimed towards children with cerebral palsy [7].

Following thematic analysis and descriptive results, all studies found that swimming does have a positive impact on FMS. All studies excluding two [10,47] found that FMS markers improve significantly because of the intervention following pre–post testing (Table 2). Pîrjol et al. [47] found that certain markers improved significantly, with overall, but not significant, improvement. Pratt et al. [10] found that there was a significant relationship (*p* = 0.01) between TGMD-2 and AMP tests, indicating swimming competency is directly related to improved motor proficiency. Similarly, Bastik et al. [5] and Rocha et al. [48] also found significant pre–post results (*p* < 0.01 [5]; *p* < 0.015 [48]); however, it is important to note that these studies included the assessment of two or more sports, which may detract away from the significance of swimming data.

Papers which assessed both male and female participants focused on the development of FMS as a result of intervention, except for one. Pratt et al. [10] identified a significant (*p* < 0.05) gender difference in that females had a higher AMP score (27.97 ± 27.90) compared to males (21.10 ± 22.70), which highlighted the potential need for a standardised assessment battery for swimming to further explore these effects. In addition, several papers did not use control groups (CG) [5,6,8,9,10,47], whereas studies that did include a CG [7,45,46,48] found significant results between them, further supporting the need for a robust swimming intervention within the curriculum, for FMS development alongside social, PA, and health benefits. There was no study included in this cohort that identified that swimming did not improve FMS development. All studies either identified that swimming improves FMS—and the data were significant such as those of Sigmundsson et al. [7] who found significant differences between the swimming and control groups following the intervention (*p* = 0.017)—or that some factors improve FMS significantly (EUROFIT test 2 (*p* = 0.002) and 4 (*p* = 0.001)) whilst others do not [47], and another identified the significant, positive relationship between swimming and FMS (*p* < 0.01) measures, but without pre–post data [10].

In addition, 7 of the 10 studies assessed pre–post data, finding significant results. Descriptive results in Table 2 represent the study by Dimitrijević [45] who found a significant increase in balance control (*p* < 0.01) and GMFM scores (*p* < 0.01) from baseline to 6-week intervention testing. Jorgic et al. [6] found a significant increase in GMFM scores post-test (*p* < 0.07). Both studies highlighted that FMS development had occurred. Eider [46] found that balance (*p* = 0.0004) and static (*p* = 0.01) and functional strength (*p* = 0.0009) all improved significantly from pre- to post-testing, again highlighting motor skill development as a result of a swimming intervention, further supported by Rocha et al. [48] who found significant increases in motor proficiency skills such as running (*p* = 0.014) and hopping (*p* = 0.009) as well as in TGMD-2 scores overall (*p* = 0.015) as a result of a 30-month intervention. Additionally, Pîrjol et al. [47] found significant improvements in some scores of motor skill development (EUROFIT tests 2 (*p* = 0.002) and 4 (*p* = 0.001)) and Moura et al. [8] found significant improvements in both basic and formal swimming skill interventions pre–post testing; the KTK test highlighted significant improvements in motor coordination (basic and formal *p* < 0.01). Moura et al.’s [9] second study also found significant results of improved motor coordination (*p* < 0.01) and improved aquatic skills (*p* < 0.01), as a result of the swimming intervention.

As a result, the main themes drawn from the papers assessed included the following: swimming improves FMS development significantly; increased frequency, intensity, time, and type (FITT) of swimming is needed generally and to improve FMS; a longer intervention duration would be beneficial; further research is needed into swimming specifically; the need for a swimming-specific assessment battery; negative comments surrounding intervention assessment tools used and intervention duration; and positives surrounding the significant effect of swimming interventions on FMS. Thematic analysis was conducted for all 10 papers and drew themes which correlated with descriptive results, including how swimming improves MS (either specific skills [47] or as a whole resulting from the outcome measure [6,8,9]), and suggested room for future research into the benefits of swimming for children and for the curriculum too.

## 4. Discussion

The objectives of this current study were (1) to assess the impact of swimming on FMS development in children aged 3–11 years, (2) to identify successful tools used to assess them independently, including TGMD-2 and AMP, and (3) to draw attention to the need for a more robust swimming curriculum generally, but particularly within the UK’s national school curriculum [13,14]. The key finding of this study is that swimming interventions were successful across the board in improving FMS, but the timeframe of interventions limited results, with the constraints of the school curriculum ultimately accountable. We appreciate that previously published works in the literature [58,59,60] have highlighted the benefits of swimming on FMS; however, the published research is scant in this specific area, specifically in 3–11-year-olds. Therefore, this current research has been influential in assessing all research within this area on 3–11-year-olds, using primary data studies measuring swimming through baseline or observational studies or a form of aquatic movement assessment and studies having used an instrument to evaluate FMS. Equally, this study did allow for the inclusion of studies which addressed sports in addition to swimming once the outcome data were found to be relevant. Across the board, there were improvements in participants’ FMS following swimming interventions; in studies where there were additional sporting interventions, it was highlighted that the swimming interventions had the highest impact on FMS development [5,48]. Out of the 10 papers assessed, only 4 used control groups [7,45,46,48], highlighting the lack of comparison had participants not been subjected to the swimming exposure in papers where a control group was not present. This was additional to other drawbacks including male-only samples [5], samples not representative of the population [45], some studies with poor or no baseline tests [48], and some studies lacking swimming-specific assessment tools [46].

### 4.1. Findings

The weight of evidence is in favour that there is a positive impact of swimming on FMS development; not only does swimming have benefits to health, fitness [16], and social and mental wellbeing [15] and provides lifesaving skills [17], it also supports development of FMS key to the overall health and development of children [58], particularly children between 3–11 years of age [59,60]. Of the 10 studies, the participants’ ages ranged from 4–10-years-old [5,7] with an average age of 7.7 years; this is an ideal range to have assessed as research suggests optimal FMS development occurs during the early years of childhood [61]. Following thematic analysis, there were clear themes within the data which coincided with the descriptive results (Table 2), specifically pre–post data. Of the 10 papers analysed, 7 documented clear pre–post data, all finding significant results regarding some or all of the test batteries; for example, Pîrjol [47], found that two of the EUROFIT assessment battery tests obtained significant data (Test 2: *p* = 0.002/Test 4: *p* = 0.001), whereas Jorgic [6] found significant increases in GMFM scores (*p* < 0.07) as a whole alongside Moura [9] who found a significant increase in KTK motor coordination scores following post-testing (*p* < 0.01). It could be argued that in studies where there were not significant increases across the board in FMS markers [46,47], these markers may indicate the limited influence swimming has on FMS development; in contrast, the inconsistent result may be due to errors within the study itself or an inappropriate or dated test battery such as the EUROFIT [56] which both Eider [46] and Pîrjol et al. [47] used, both finding some results more significant than others. However, recent research [62] highlighted the EUROFIT as a reliable method for assessing the effects of swimming on FMS in secondary school-aged pupils; however, this study only used a select few of the EUROFIT test batteries which may exclude those tests that these studies did include. It did, however, highlight the need for swimming to be implemented more thoroughly in the national curriculum in the UK and found significant results pointing towards the same conclusions as the data in this current study, suggesting that swimming does impact motor skill development. Additionally, the inconsistent EUROFIT data may be due to a lack of research into its application in aquatic settings; recent research highlights its test–retest reliability but also notes a lack of research supporting its use [63], especially as there is no research into its application in a swimming setting. This is further supported by research suggesting that modifications to the test battery may be needed for application of specific tests within the battery as some hold more reliability than others [64], which would further support the significance of some tests but not others. In contrast to this, other research has identified that the EUROFIT has test–retest reliability but also noted that other studies (not related to swimming/FMS) had similar problems with the significance of data for some tests and not others, concluding inconsistency in test methods between each test [65].

In summary, throughout the process of data collation for this study, assessing results of studies that do not use a consistent testing battery increases the risk of influences such as researcher bias, reliability of test methods, as well as comparability, with a meta-analysis not possible in this circumstance. It is hard to ignore the importance of a consistent and streamlined testing method for FMS and swimming as independent topics, to allow for the effects of an intervention such as swimming on FMS to be clearly and easily identified. It may be important to assess if one test works better than another in future research, to identify the best test for FMS. A further future recommendation to encourage the need for a universal and reliable test battery for FMS, and swimming independently, is essential as studies have further reported limitations of the ceiling effect which may reduce significant results [66] as well as the only modern and reliable test method suggested for swimming being the AMP, only newly developed by Pratt et al. [10] and still requiring further research. In terms of future research, it would be appropriate to complete a meta-analysis of each of the tools used to assess FMS and swimming and then compare them for more robust findings to identify which tool would be more appropriate when assessing FMS and swimming competency.

In the two papers where swimmers were compared to children participating in other sports to assess FMS development, both [5,48] used the TGMD-2 test, finding that those subjected to a swimming intervention scored higher in these tests compared to those of other sports or control groups. More specifically, in Bastik et al.’s research [5], the highest overall TGMD-2 score of the group was 102.70 ± 9.3 for swimming, whereas the next highest was for table tennis (98.75 ± 10.7), and the lowest score was from the court tennis group (80.00 ± 12.6), with both having higher variance between the subjects within their groups also. In contrast to this, Rocha et al. [48] found that both swimming and football provided long-term benefit to FMS development; although swimming scored higher (124.81 ± 7.83) compared to the control group in post-testing at 30 months (99.18 ± 12.59), it did not score higher compared to the football intervention (133.55 ± 6.67), which conflicts with Bastik et al. [5], even though their football intervention did score high in post-testing, which may be due to differences in the test methods used by each research group, this helps to confirm the fact that swimming is one of the most impactful sports contributing to FMS development. However, it does raise questions as to whether swimming is the most impactful sport for FMS development, as conflicting research suggests motor skill development should not be bound by sports participation [67], whereas other research [2] coincides with Bastik et al. [5] and Rocha et al. [48], supporting that sport is a key development tool for FMS; research explicitly stating swimming is the best sport for FMS development is limited if not non-existent, but this study shows that swimming improves motor skill development (Table 2), as do other sports including football [48] and gymnastics [2], for example, and highlights a need for future research specifically assessing the benefits of swimming to FMS development using modern, reliable, robust, well-researched assessment tools for both swimming and FMS. In support of this, thematic analysis identified a subtheme which highlighted the need for swimming-specific research into the effects on FMS, and this is supported by the lack of papers included in the study due to irrelevance and not meeting the inclusion criteria, with many papers excluded for including other sports for interventions and not fully documenting the specific effects of a swimming intervention on FMS. As previously stated, our inclusion criteria ensured the inclusion of research that analysed swimming, either observationally or through an aquatic movement assessment, and FMS, using an instrument to evaluate it; however, limited research surrounding this area and limited robust assessment tools for swimming meant minimal studies were included.

What was also highlighted was that many of the papers included in the final analysis made comment on the intervention time being too short to see the full effects of swimming [6,8,45] as well as not allowing time to develop full skill mastery [8,9,46]. This was further supported by research supporting that the development of skills is hindered by the constraints of the school curriculum limiting time and duration of swimming lessons [68]; equally, candidates for the sample were most commonly found through local schools and clubs due to convenience. The sample consisted of 10 studies that met the inclusion criteria for this review and, of these, only two were from the UK, emphasising the scant data within this area and country. This is surprising, since the literature has strongly suggested that swimming can be beneficial to children’s general health, mental health, coordination, and balance [15,16,18] and is currently the only compulsory sport in the UK national curriculum [17]. Considering that research supports the positive impact of swimming interventions on FMS development [8,9] and children who are underdeveloped in terms of FMS are associated with a higher risk of inactivity and sedentary behaviour [34], the persistent paucity of research on swimming and FMS is concerning and requires urgent research attention in the future.

A large proportion of negative themes within this current study surrounded the lack of research and FITT criticisms as well as future recommendations surrounding the need to increase both [7,9,45]; this further supports the need to encourage future research into swimming-specific interventions associated with FMS development. Many participants were selected through convenience sampling within the schools they attended, where swimming lessons were part of their curriculum [8]; again, a major theme emerged in that this FITT drawback was due to research being bound by the constraints of the curriculum’s time allocation for swimming [8,10,46] and further highlights the need for the curriculum to be addressed regarding swimming as well as increasing research on this topic. Additional findings by Pratt et al. [10] were the only to address gender differences, finding that females had significantly (*p* = 0.05) higher aquatic competence scores (27.97 ± 27.90) compared to males (21.10 ± 22.70) and higher locomotor scores compared to males (F, 14.9 ± 0.4/M, 14.2 ± 0.4; *p* = 0.05). However, Lubans et al. [59] found that males were better than females in terms of object control which conflicts with some of this research. van Beurden et al. [69] identified that males were better at both locomotor skills tests as well as object control which conflicts with data found by Pratt et al. [10]. This conflicting evidence highlights the need for further research specifically looking at gender differences or the gender-specific effects of swimming on FMS, as this may affect the way a programme is delivered or assessed, especially as girls start puberty at an earlier age than boys on average [70] and may feel the benefits of this through their skills and FMS development. It is hard to pinpoint which gender is better than the other regarding FMS skills relating to a swimming intervention, again, due to the lack of research in this area, as well as Pratt et al.’s [10] AMP assessment tool being a new process which is yet to be assessed for test–retest reliability, requiring further research. This further highlights the requirements for a standardised assessment battery for swimming and the need for a robust swimming intervention within the curriculum for FMS development, alongside social, PA, and health benefits.

### 4.2. Review of Data

The study by Bastik et al. [5] found significant data in support of swimming improving FMS development; however, the study did highlight some limitations. The research did not include the use of a control group, making it harder to identify the true impact of a swimming intervention; studies such as that by Sigmundsson et al. [7] identified the significant difference between the swimming and control groups (*p* = 0.017) and, therefore, the true benefit of a swimming intervention, whereas Bastik et al. [5] could only highlight the benefits of swimming compared to additional sports, although swimming scored highest following TGMD-2 testing. Unfortunately, several other studies did not utilise a control group, making it harder to compare the effects to intervention exposure [6,8,9,10,47], something which a control group would help to demonstrate. Furthermore, the study by Bastik et al. [5] lacked baseline testing, which further makes the true benefits of an intervention hard to assess, reducing result robustness, especially when comparing against multiple other sports. This also does not account for any prior experience a child may have in an intervention: some may have had swimming lessons before whereas others may have never played table tennis, putting the swimming group at an unfair advantage and giving further false representation of results. To further this, Rocha et al. [48] compared swimming to a football intervention, also lacking baseline testing, raising the same issues; however, Rocha et al. [48] did test incrementally but still did not consider influences that pre-testing would have ruled out. For example, Eider [46] used pre–post testing, and this allowed significant improvements to be identified which Bastik et al. [5] and Rocha et al. [48] could not, as well as ensuring the validity of results. In comparison to this, Rocha et al. [48] conducted further research which did utilise baseline testing which proved to highlight significant results in development of skills as a result, further highlighting the importance of baseline testing.

In addition to this, the research by Bastik et al. [5] used a male-only participant sample, which reduces the generalisability and does not represent the target population; FMS does not only apply to the male gender. Unfortunately, the male-only sample was used in two other studies [46,48], highlighting the same concerns regarding practical application. The lack of generalisability of representative samples was a common theme amongst the studies used for this review. Many studies’ participants were not representative of the age-appropriate population [5,8,10,45] and also excluded SEND groups [48] who receive proven benefits from aquatic therapy and swimming to their health, wellbeing, and motor function [4,45]. A further limitation identified was the use of small participant samples further reducing the reliability of results [6,45,47].

Research by Dimitrijević et al. [6] amongst many other papers highlighted the limitations of a short intervention. In fact, 9 out of 10 studies identified that the intervention was too short in terms of time span, with interventions usually taking place over weeks to months, with only one study taking a longitudinal approach [48]. Many papers commented this was due to the constraints of the school curriculum [9,47], whilst others suggested future research should consider looking at the benefits of a swimming intervention over a longer period [6,8,45]; this could help to support the requirement for swimming in the UK national curriculum during key stages 1 and 2 [13,17]. Longitudinal studies in relation to child development are essential in order to obtain a clear picture of the development process and influences which would influence factors such as the school curriculum, developing a more accurate picture [71] of the true influences of an intervention such as swimming. This is supported by previous research [72] that assessed the benefits of FMS development throughout childhood, assessing children in 2000 and again in 2008, finding that participation in sports greatly develops FMS but this is influenced by length of participation, highlighting the importance of future longitudinal studies on the effects of swimming in FMS development for longer periods (1 year or more). In addition to this, it should be considered that it is not always possible to conduct long-term research due to the convenience and constraints of the school curriculum [9,47] and, fortunately, all studies found significant data within some/all of their tests, such as the study by Moura et al. [9], who found swimming improved motor skills significantly (*p* < 0.01). The clear curriculum constraints highlight a need for future research addressing this pitfall, to evidence the notion that longer participation in swimming through the curriculum may further support child development. Equally, this literature review found that only two studies were relevant to the UK, therefore supporting the requirement for a more robust swimming curriculum generally, but particularly within the UK national curriculum. A further limitation was the poor quality of the test methods used; for example, the EUROFIT test battery is slightly dated [56] and conflicting research suggests the test may contribute to fluctuating significance between each test battery [63,64]. Research by Eider [46] highlighted this as well as that by Pratt et al. [10] and research not included in this study [63,64,65]. Similarly, the lack of a valid and reliable test method for swimming became apparent, as only three studies utilised a swimming test method, such as the AMP [10] or the WOTA-2 [6,45], and two used an observation checklist [8,9].

In contrast, despite the many limitations from various papers, the studies did meet sufficient scores when using the MMAT assessment tool [44] and did provide data that met all inclusion and exclusion criteria. Adjustments were made to inclusion criteria such as including SEND-participant papers, due to the lack of research in swimming and FMS paired with limited standardised test methods, especially swimming-specific ones [10]. This calls for the need for future research into identifying a robust, reliable, and valid assessment tool for swimming development as well as standardising the FMS assessment tools used. These findings also press the need for future research into the effects of swimming on FMS development, especially over a longer period of time, and constraints of the UK national curriculum need to be addressed, suggesting policy and curriculum revision to benefit the development of FMS for children throughout childhood, not just for KS1/KS2 [13]. This would no doubt increase the need for additional funding to support development.

### 4.3. Strengths and Limitations of the Current Study

This study has addressed the specific objectives it set out to investigate, namely (1) regarding all studies included in the synthesis, there is an impact of swimming on FMS development and it is positive and (2) some tools were more reliable than others: the TGMD-2 test and GMFM-88 indicated significant results for the papers they were used in, whereas methods such as SMAB were highlighted as dated [7], and the EUROFIT was highlighted as inconsistent and, therefore, less successful in comparison to other tests [46,47]. In addition, there is an apparent lack in standardised testing methods for swimming, with the emergence of a new testing method addressed by Pratt et al. [10]; there needs to be further research investigating this as well as possible alternatives. Other strengths of this study include using an explicit bias-reducing assessment tool (MMAT) [44], inclusion of reliable sources and research, and a comprehensive review in line with PRISMA methodology with no selective preferences and an open approach following standardised and reliable review methods utilised [42,43] to ensure lack of bias. Most importantly, the findings of this research positively highlight the benefits to child development throughout childhood, not just in a specific age group, and swimming is the only sport providing physical activity as well as lifesaving skills [17]. Therefore, it is important to carry this on in future research, further exploring the benefits of swimming on FMS development, creating a standardised robust assessment battery for swimming, and pushing to address the UK national curriculum and its wording.

With every study there are limitations; in this case, they have arisen from a lack of research addressing this topic, as well as the fact that papers that did address the effects of swimming on FMS also featured imbalances in the numbers of male and female participants, did not include control groups, and did not use the same testing methods, and the number of papers meeting the inclusion criteria were limited. What this meant was a meta-analysis was not possible, which may have provided deeper insight into the data collated (Table 2). In addition, the quality of a systematic literature review is dependent on the quality of the papers identified for synthesis [73] which, in this case, may have held some influence. That said, the papers synthesised in this study have been influential in supporting the significant positive impact of swimming on FMS.

### 4.4. Future Recommendations

In response to the final objective to (3) provide recommendations appropriate to the UK national curriculum based on the findings of this study, there are many recommendations due to the lack of research in this specific field. Future recommendations include the need for future research specifically addressing a standardised, robust test battery for swimming assessment building on Pratt et al.’s [10] research. Future research into standardising the assessment tools used for FMS to reduce inconsistency and reports of the ceiling effect is recommended [67]. Future research investigating the long-term effects of swimming on FMS development is recommended, meaning there needs to be increased FITT recommendations [9,45] not bound by the constraints of the school curriculum [19] which will further help to address the concerns of the current UK national curriculum [13]. Other suggestions include future research looking at the gender differences in FMS development following a swimming intervention, as only one of ten studies highlighted this. Finally, future recommendations include re-assessment of the UK national curriculum, looking at specifying where funding is spent [13,14,19], increasing funding, and increasing the length of time spent teaching swimming skills within the school curriculum; this would require revision of the national curriculum [20], providing more specific detail regarding how swimming is delivered (FITT) and assessed.

## 5. Conclusions

To our knowledge, this is the first SLR to investigate the effects of swimming on FMS development and the first study to investigate this by specifically addressing the UK national curriculum and its constraints; it is evident there is more research needed regarding swimming and FMS development. This research supports previous research findings in that swimming does improve FMS development significantly. Increased frequency, intensity, time, and type of swimming is needed generally, and to improve FMS, a longer intervention duration and research into gender differences would be beneficial in this area. Equally, this research discovered that some assessment tools proved more robust than others and that future research is required to establish standardised assessment tools for both swimming and FMS independently. There are also recommendations for revision of the national curriculum to benefit child development more broadly, ensuring that children have access to swimming for motor skill development and essential lifesaving skills, influencing many aspects of a child’s development [8,9,10,18] besides their ability to swim competently.

## Figures and Tables

**Table 1:** MMAT Quality Assessment Outcomes.

Author and Year of Publication	MMAT Score
Bastik et al., 2012 [5]	6
Dimitrijević et al., 2012 [45]	5
Eider et al., 2015 [46]	6
Jorgic et al., 2012 [6]	6
Moura et al., 2021 [8]	7
Moura et al., 2022 [9]	7
Pîrjol et al., 2017 [47]	5
Pratt et al., 2021 [10]	7
Rocha et al., 2016 [48]	6
Sigmundsson et al., 2010 [7]	6

**Table 2:** Swimming and FMS Study Descriptive Results.

Author	Country	Setting	Sample Size (M/F)	Mean Age (Years)	Study Design	Outcome Measure	Overall Findings
Bastik et al. (2012) [5]	Turkey	Local sports club	120 males (M)	10.19 ± 0.24	Experimental non-randomised controlled trial	(1) TGMD-2	(1) Participants who were swimmers compared to other children/non-swimmers scored the highest in the TGMD-2 test (102.7 ± 9.3) (lowest score = court tennis, 80.0 ± 12.6); (2) All tests from TGMD-2 showed significantly improved scores for the swimmers compared to all other children/non-swimmers (locomotor subtest, 40.1 ± 2.9 (*p* < 0.001)) (object control, 62.5 ± 8.3 (*p* < 0.001)).
Dimitrijević et al. (2012) [45]	Serbia	Local sports centre	27 (10 females (F), 17 M)	9.56 ± 2.37	Experimental randomised controlled trial	(1) GMFM-88 (2) WOTA-2	(1) Study observed a significant improvement in balance control from the baseline (B) test to 6-week (6W) WOTA-2 testing (B, 20.71 ± 10.82/6W, 31.93 ± 9.10 (*p* < 0.01)) highlighting the improvement in MS due to aquatic skill development; (2) There was a significant improvement in GMFM scores in control from baseline to 6W testing (B, 73.53 ± 25.63/6W, 77.92 ± 23.63 (*p* < 0.01)); (3) Most effective after 6-week intervention compared to 9-week intervention (significant results compared to baseline at 6W compared to 9 weeks); (4) Highlights the benefit of aquatic PA on children’s FMS development.
Eider (2015) [46]	Poland	Local swim club’s pool	94 M	7	Observation, longitudinal, non-randomised, cohort study	(1) eight tests from EUROFIT Test Battery (Speed; Explosive Power; Balance; Flexibility; Functional Upper Body Strength; Static Strength; Torso Strength; Agility Run)	(1) Regular participation in sports (swimming) positively impacts MS development; (2) There were significant improvements in all tests because of swimming compared to control group (CG); (3) The most improved areas for the swimming group included balance (pre, 8.0 ± 1.1/post, 4.0 ± 1.2 (*p* = 0.0004)) and static (pre, 2.5 ± 0.9/post, 3.5 ± 1.0 (*p* = 0.01)) and functional (pre, 10.7 ± 5.8/post, 13.7 ± 6.2 (*p* = 0.0009)) strength.
Jorgic et al. (2012) [6]	Serbia	Clinical lab within university	7 (3 F, 4 M)	9.42 ± 1.25	Experimental pilot, non-randomised controlled trial	(1) GMFM-88 (2) WOTA-2	(1) Significant difference between initial and final testing of GMFM (pre, 89.47% ± 12.53/post, 91.11% ± 10.66 (*p* = 0.07)); (2) Proven improvement in FMS (walking, jumping, and gross MS) due to the swimming condition.
Moura et al. (2021) [8]	Portugal	School pool	31 (15 F, 16 M)	8.00 ± 0.86	Experimental, randomised controlled trial	(1) Aquatic observation checklist (2) KTK	(1) Regular swimming led to significant improvement in aquatic readiness and motor coordination, including skills like coordination, kicking, and breath control (basic—2.29 ± 1.05 pre vs. 3.06 ± 1.03 post (*p* < 0.01)/formal—2.07 pre vs. 2.79 ± 0.98 post (*p* < 0.02)); (2) Motor coordination significantly improved in both conditions of swimming (basic CG: 130.18 ± 37.71 pre vs. 162.71 ± 40.40 post (*p* < 0.01)/formal CG 135.57 ± 37.45 pre vs. 172.64 ± 33.17 post (*p* < 0.01)), highlighting how swimming stimulates motor learning via KTK test.
Moura et al. (2022) [9]	Portugal	School pool	50 (26 F, 24 M)	8.34 ± 1.10	Experimental, randomised controlled trial	(1) Aquatic observation checklist (2) KTK	(1) Swimming improved aquatic skills significantly (31.40 ± 12.89 points pre vs. 46.9 ± 10.73 points post (aquatic observation)); (2) Swimming also improved motor coordination significantly (110.23 ± 20.52 points pre vs. 147.23 ± 30.10 points post (KTK), where *p* < 0.01); (3) Regular swimming is highly beneficial for motor development and water safety.
Pîrjol et al. (2017) [47]	Romania	Afterschool field setting	10 (5 F, 5 M)	7.10 ± 0.73	Experimental, non-randomised controlled trial	seven tests from (2) EUROFIT (Explosive Power; Flexibility; Upper Body Strength; Lower Body Strength; Core Strength; Back Strength; Agility Run)	(1) Each individual had improved from pre- to post-testing; (2) Each test showed some improvement due to swimming intervention; (3) EUROFIT test 2 significantly improved (24.5 pre vs. 27 post, *p* = 0.002); test 4 also significantly improved (108.6 pre vs. 119.8 post, *p* = 0.001).
Pratt et al. (2021) [10]	United Kingdom	Cohort from five primary schools	201 (105 F, 96 M)	7.80 ± 0.63	Experimental, non-randomised controlled trial	(1) TGMD-2 (2) AMP	(1) AMP proven as a valid method for assessing aquatic competence; (2) Swimming practice during childhood contributes to higher motor development; (3) Clear positive impact of swimming on dryland MS; importance of swimming in the curriculum is evident; (4) Females shown to have greater aquatic competence (27.97 ± 27.90) than males (21.10 ± 22.70), where *p* = 0.05, as well as higher locomotor scores (F, 14.9 ± 0.4/M, 14.2 ± 0.4 (*p* = 0.05)); (5) Males better at object control (14.5 ± 0.6) compared to females (13.6 ± 0.6); (6) No significant results in relation to body mass and aquatic competence; (7) AMP and TGMD-2 scores shown to have significant relationship (*p* = 0.01) indicating those who are more competent at swimming have better motor proficiency; (8) Children with significantly high aquatic competence had higher motor proficiency (*p* = 0.001).
Rocha et al. (2016) [48]	Portugal	School pool and football pitch	33 M	4.80 ± 0.5	Experimental longitudinal, non-randomised controlled trial	(1) TGMD-2	(1) “Positive impact of swimming and soccer participation on motor proficiency.”; (2) TGMD-2 testing in the swimming group observed significant increase between 5–30-month tests (T5, 101.91 ± 19.82/T30, 124.81 ± 7.83 (*p* = 0.015)); (3) Swimming saw significant increase in motor proficiency in running (T5, 5.73 ± 1.79/T30, 8.00 ± 0.00 (*p* = 0.014)) and hopping (T5, 4.27 ± 4.15/T30, 9.09 ± 1.64 (*p* = 0.009)) locomotor tests; (4) Swimming and soccer both result in motor proficiency development, but swimming provides ongoing development.
Sigmundsson et al. (2010) [7]	United Kingdom	Local leisure centre	38 (18 F, 20 M)	4.72 ± 0.24	Experimental, Non-randomised controlled trial	(1) SMAB	(1) Data did not highlight many significant data between swimming and CG; (2) There were significant differences following the experiment between both CG and swimming (SW) groups (SW, 0.02/CG, 0.04/*p* = 0.017), indicating swimming has a significant impact on FMS development.

Key: TGMD-2—Test of Gross Motor Development-2; GMFM—88—Gross Motor Function Measurement—88 Tests; WOTA-2—Water Orientation Test Alyn 2; KTK—Körperkoordinationtest Für Kinder test; AMP—aquatic movement protocol; SMAB—Standardized Movement Assessment Battery.

## Data Availability

The data presented in the study are available in the article.
